# Identification of Bias in Ordering Further Imaging in Ethnic Groups With Indeterminate Ultrasound for Appendicitis

**DOI:** 10.7759/cureus.28109

**Published:** 2022-08-17

**Authors:** Puja Desai, Lindsey Haut, Brian Wagers, R. Lane Coffee, Heather Kelker, Michael Wyderko, Elisa J Sarmiento, Jessica Kanis

**Affiliations:** 1 Emergency Medicine, Indiana University School of Medicine, Indianapolis, USA; 2 Pediatrics, Indiana University School of Medicine, Indianapolis, USA; 3 Internal Medicine, University of Central Florida College of Medicine, Orlando, USA

**Keywords:** emergency department, imaging, racial disparities, bias, appendicitis

## Abstract

Background

Recent studies have shown a higher incidence of complications from acute appendicitis in Hispanic populations. Hispanic ethnicity alone has been shown to be a risk factor. In contrast, one study found little evidence of racial disparities in complication rates. The objective of this study was to identify physician bias regarding whether ethnicity drives further testing after initial radiologic imaging has been obtained in the evaluation of appendicitis in our pediatric emergency department (PED). The use of computed tomography (CT) scan in the diagnosis of appendicitis was compared between Hispanic versus non-Hispanic populations when ultrasound (US) was indeterminate.

Methodology

This is a retrospective cohort study of Hispanic and non-Hispanic patients aged 2-18 who presented to the PED with right lower quadrant abdominal pain over a one-year period (January 1, 2017 to December 29, 2017). Both groups were subdivided into positive, negative, or indeterminate US findings for appendicitis. Each subgroup was analyzed based on those who had CT imaging done.

Results

A total of 471 ultrasounds were performed, 162 Hispanic and 309 non-Hispanic patients. Indeterminate US scans were documented in 90/162 (56%) Hispanic versus 155/309 (50%) non-Hispanic patients. Of those with indeterminate US scans, 30% Hispanic versus 32% non-Hispanic patients received CT scans. Negative US scans were documented in 54/162 (33%) Hispanic versus 102/309 (33%) non-Hispanic patients. Of those with negative US scans, 7% Hispanic versus 5% non-Hispanic patients received CT scans. Chi-square analysis comparing both the proportion of CT scans received for indeterminate US scans (p=0.71) and negative US scans (p=0.52) showed no statistical significance.

Conclusions

There was no significant difference in the number of CT scans ordered for indeterminate US scans between Hispanic and non-Hispanic patients. One can infer that there is no inherent bias toward ordering advanced imaging in Hispanic children based on ethnicity alone.

## Introduction

Appendicitis is one of the most common emergencies in childhood, and the vast majority are treated surgically [1-3]. Approximately 7% of children presenting to emergency rooms with abdominal pain have acute appendicitis [4]. In a recent study utilizing disparities analytic files from 2015, 45% of pediatric appendicitis cases exhibited perforation as a major complication [2]. Hispanic and African American populations demonstrate an increased incidence of perforated appendicitis [3]. Ladd et al. reported increased rates of complications in Hispanic versus African American children, regardless of timing of diagnosis [5]. Guagliardo et al. reported that while controlling for confounders such as socioeconomic factors, Hispanic ethnicity by itself was a risk factor for complications [6]. In contrast, two studies have found little evidence of racial disparities in perforated acute appendicitis rates [2,7].

It has been found that ethnic minority groups are at increased risk for morbidity and mortality in several disease processes [5]. With this information, medical research has previously emphasized the role of cognitive biases in the diagnostic process [8]. Anchoring bias is where one piece of initial information is weighed heavily and relied upon in decision making despite other information present that may offer other conclusions [8]. While cognitive bias is an emerging area of interest in medical literature, there are limited studies in the pediatric population that identify potential biases in the diagnosis of appendicitis.

The diagnosis of appendicitis remains a challenge. It has been shown that ultrasonography is more accurate than clinical assessment in diagnosing acute appendicitis [9]. Although computed tomography (CT) scan remains the most accurate modality to rule out acute appendicitis [9], the risks associated with its use such as radiation exposure, increased length of stay, and cost must be weighed [10,11]. Therefore, ultrasonography remains the first-line imaging modality in the evaluation of appendicitis given that it is safe, quick, and relatively inexpensive to use [9,12].

Disparities in diagnostic imaging for appendicitis among ethnic groups have been studied. Wang et al. found that minorities and low-income children had lower rates of radiologic imaging including CT scans for appendicitis [13]. A multicenter emergency department (ED) study of pediatric visits for abdominal pain found variation in the use of advanced imaging across patient ethnicity and insurance status, specifically that minority patients and those not privately insured were less likely to receive advanced imaging for abdominal pain [14]. Meanwhile, Caperell et al. performed a retrospective study investigating demographic and clinical factors of children who presented to the pediatric emergency department (PED) with abdominal pain and found that no racial differences exist in the evaluation, treatment, and disposition of these children [15]. Previous studies demonstrate conflicting evidence to suggest disparities in quality of healthcare for children of different ethnicities. In addition, there are few published studies that identify physician implicit bias in imaging disparities in the pediatric population. This study, which was a sub-analysis of a larger data set, sought to identify bias regarding whether ethnicity drives further testing after initial radiologic imaging has been obtained in the evaluation of appendicitis in our PED. Thus, the study aim was to compare the use of CT scan in the diagnosis of pediatric acute appendicitis between Hispanic versus non-Hispanic populations when ultrasound (US) is indeterminate.

This article was previously presented as a meeting abstract at the 2021 Society for Academic Emergency Medicine Conference on May 12, 2021.

## Materials and methods

This study was conducted at Riley Hospital for Children in Indianapolis, Indiana. Riley Hospital for Children is an academic quaternary care pediatric hospital with an emergency department that averages 50,000 visits per year.

Data were primarily collected and managed using REDCap (Research Electronic Data Capture) hosted at Indiana University [16,17]. This study was evaluated through the Institutional Review Board (IRB) at Indiana University and was considered exempt (protocol 1803688299).

A retrospective cohort study of patients aged 2-18 who presented to our PED with right lower quadrant abdominal pain and received a US scan for the diagnosis of appendicitis over one year from January 1, 2017 to December 29, 2017. Patients were identified via query of our institutional Electronic Medical Record (EMR). Baseline demographics obtained included age, sex, race, and ethnicity which were gathered by our registration team. To capture ethnicity, patients were determined to be Hispanic or non-Hispanic by two chart reviewers using a rater perceived Hispanic name. Both raters were blinded to one another’s designation. These judgments were interrogated using inter-rater reliability with a kappa analysis.

Charts were further reviewed for laboratory and imaging obtained (US and CT) and results of imaging. Three outcomes with standard diagnostic criteria defined ultrasound results: positive (findings are consistent with appendicitis), negative (the appendix is seen in its entirety and is normal), or indeterminate (the appendix was not seen in its entirety, and there are no secondary findings of appendicitis). Subgroup analyses were based on those with further radiological imaging (CT).

Descriptive statistics were used, with data presented using percentages and chi-square tests for categorical variables and means and t-tests for continuous variables. P-values less than 0.05 were considered statistically significant. All statistical analyses were performed using SAS Version 9.5 (SAS Institute Inc., Cary, NC, USA). Kappa analysis was performed for agreement on perceived ethnicity designation with values of 0.01-0.20 considered none to slight, 0.21-0.40 fair, 0.41-0.60 moderate, 0.61-0.80 substantial, and 0.81-1.00 almost perfect agreement.

## Results

A total of 471 ultrasounds were performed to evaluate for acute appendicitis, of which 57 were positive, 93 were negative, and 321 were indeterminate. Table 1 shows the baseline demographics of our study population including age, sex, and ethnicity. The majority of ultrasounds were performed on non-Hispanic children compared to Hispanic children, 309 (65%) versus 162 (35%), respectively. Table 2 demonstrates testing outcomes for our patient population. A similar proportion of Hispanic patients had indeterminate ultrasounds (90/162, 56%) compared to non-Hispanic patients (155/309, 50%). Of the patients with indeterminate ultrasounds, approximately one-third of all patients received subsequent CT scans, 30% of Hispanic patients versus 32% of non-Hispanic patients. Of the 27 Hispanic patients who received CT scans, 26% were positive for appendicitis, 63% were negative, and 11% were indeterminate. Of the 50 non-Hispanic patients that received CT scans, 24% were positive for appendicitis, 62% were negative, and 14% were indeterminate.

**Table 1:** Demographics of the study population.

Variable	n=471
Age, mean	2-18 years, 9.10 years
Sex-male, n (%)	186 (45.8%)
Sex-female, n (%)	285 (54.2%)
Hispanic, n (%)	162 (34.4%)
Non-Hispanic, n (%)	309 (65.6%)

The majority of ultrasounds obtained between both Hispanic and non-Hispanic patients were indeterminate (53%) compared to negative (33%). Further analysis of the negative US results was conducted. Negative US scans were documented in 54/162 (33%) Hispanic patients versus 102/309 (33%) of non-Hispanic patients. Of Hispanic patients with negative US scans, 4/54 (7%) received CT scans and all had negative results. Of non-Hispanic patients with negative US scans, 5/102 (5%) received CT scans with 1/5 (20%) positive for appendicitis, 2/5 (40%) negative, and 2/5 (40%) indeterminate.

**Table 2:** Indeterminate ultrasound scan results. *Chi-square test used to estimate P-value; n.s. = not significant

	Hispanic	Non-Hispanic	P-value
Indeterminate ultrasound scan results	90/162 (56%)	155/309 (50%)	n.s.*
Follow-up abdominal computed tomography (CT) scan completed	27/90 (30%)	50/155 (32%)	n.s.*
CT results			
Positive	7 (26%)	12 (24%)	n.s.*
Negative	17 (63%)	31 (62%)	n.s.*
Indeterminate	3 (11%)	7 (14%)	n.s.*

Chi-square analysis comparing the proportion of CT scans done for indeterminate US scans between both groups demonstrated no statistical significance (p=0.71). Chi-square analysis comparing the proportion of CT scans done for negative US scans between both groups also demonstrated no statistical significance (p=0.52). A Kappa value of 0.93 suggested an almost perfect level of agreement (95% CI 0.89-0.96, Standard error = 0.02).

## Discussion

In this study, charts of Hispanic and non-Hispanic patients presenting to the PED with right lower quadrant abdominal pain were retrospectively reviewed. Basic demographic information was gathered. A kappa analysis was performed for agreement on perceived ethnicity designation and demonstrated an almost perfect level of agreement. Ultrasound results for pediatric appendicitis among Hispanic and non-Hispanic patients were categorized into three groups: positive, negative, or indeterminate per previously described criteria. Results indicate that the majority of ultrasounds obtained for both Hispanic and non-Hispanic children were indeterminate. We believe the rate of indeterminate results was directly related to body habitus as determined in a separate analysis currently under consideration for publication. We further analyzed patients who had indeterminate US scans for appendicitis to evaluate our bias in ordering advanced imaging with CT based on ethnicity alone. We found similar utilization rates of CT scans in Hispanic and non-Hispanic groups who had indeterminate US scans, therefore we did not identify bias in ordering advanced imaging based on perceived ethnicity. Despite several studies emphasizing that Hispanic children have increased risk of complicated appendicitis, we found that our PED did not pursue CT scans unless indicated.

Healthcare inequalities have been identified in the literature. This evolving area of research aims to recognize factors that contribute to inequalities in the care and outcomes of children, with emphasis on exploring the role of implicit and explicit bias in healthcare. Implicit biases are unconscious attitudes and beliefs that may influence behaviors such as nonverbal communication, physician perceptions and clinical assessments about patients, and decisions about patient management [18]. Research on implicit bias in healthcare settings have focused primarily on adults. There is an emerging evidence in pediatrics demonstrating inequalities and recent studies documenting implicit bias towards children.

There are various studies conducted in PEDs that have established racial and/or ethnic disparities in analgesic management for children presenting with acute abdominal pain and appendicitis [19,20]. Drapkin et al. found a correlation between the triage chief complaints and rates of missed appendicitis, identifying potential anchoring bias as the underlying cause of diagnostic medical errors [1]. This tendency to concentrate on specific aspects of a presentation too early in the diagnostic process and subsequent failure to adjust is often an unconscious act [8]. Raphael et al. explained that implicit biases function to the disadvantage of vulnerable populations, including racial and/or ethnic minorities [21]. Many studies have shown that implicit biases may be heightened in emergency departments due to stressors such as high acuity, complex decision-making, time pressure, workflow interruptions, and fatigue which can pose challenges in delivering high quality, effective care. These factors may exacerbate physician reliance on cognitive biases [22,23]. Understanding cognitive bias is the initial step in identifying potential biases, subsequently allowing for awareness of the effects of biases, with the aim to decrease the rates of diagnostic errors.

Study limitations are those to be expected with a retrospective chart review including the completion of the data entered in REDCap. This study is a retrospective chart review of an administrative database, and our analysis depends on the integrity of the clerical data entry. Although we have no suspicion of the quality of our documentation, we do recognize that such omissions can skew results. In addition, this study analyzed data from a single pediatric institution and may not be generalizable to other settings. Other confounding factors may have played a role in the decision to order advanced imaging in this cohort, such as PAS score, parental wishes, or based on surgical specialty recommendations.

## Conclusions

To our knowledge this is the first study exploring physician bias in ordering advanced imaging in ethnic groups with indeterminate US for appendicitis. Our results demonstrating that there was no significant difference in utilization of CT scans for indeterminate US scans between Hispanic and non-Hispanic populations. This suggests there is no inherent bias toward ordering advanced imaging in Hispanic children based on perceived ethnicity alone. Implicit bias and its effects on medical care and caregivers is an area of medicine that needs continued exploration, training, and awareness to improve patient safety and outcomes, and to minimize diagnostic errors. The PED is a unique and vital environment to examine the impact of bias on racial disparities. Further research exploring the relationship between patient ethnicity, physician bias, and cognitive stressors in the emergency setting is crucial in understanding the disparities that exist and how to improve healthcare and outcomes of children.
